# A Putative ABC Transporter Permease Is Necessary for Resistance to Acidified Nitrite and EDTA in *Pseudomonas aeruginosa* under Aerobic and Anaerobic Planktonic and Biofilm Conditions

**DOI:** 10.3389/fmicb.2016.00291

**Published:** 2016-04-01

**Authors:** Cameron McDaniel, Shengchang Su, Warunya Panmanee, Gee W. Lau, Tristan Browne, Kevin Cox, Andrew T. Paul, Seung-Hyun B. Ko, Joel E. Mortensen, Joseph S. Lam, Daniel A. Muruve, Daniel J. Hassett

**Affiliations:** ^1^Department of Molecular Genetics, Biochemistry and Microbiology, University of Cincinnati College of MedicineCincinnati, OH, USA; ^2^College of Veterinary Medicine, University of Illinois at Urbana-ChampaignUrbana, IL, USA; ^3^Diagnostic and Infectious Diseases Testing Laboratory, Cincinnati Children's Hospital Medical CenterCincinnati, OH, USA; ^4^Department of Molecular and Cellular Biology, University of GuelphGuelph, ON, Canada; ^5^Department of Medicine, University of CalgaryCalgary, AB, Canada; ^6^Department of Research Services, Cincinnati Veteran's Association Medical CenterCincinnati, OH, USA

**Keywords:** *Pseudomonas aeruginosa*, acidified nitrite, EDTA, biofilms, ABC transporter permease

## Abstract

*Pseudomonas aeruginosa (PA)* is an important airway pathogen of cystic fibrosis and chronic obstructive disease patients. Multiply drug resistant *PA* is becoming increasing prevalent and new strategies are needed to combat such insidious organisms. We have previously shown that a mucoid, *mucA22* mutant *PA* is exquisitely sensitive to acidified nitrite (${\text{A-NO}}_{2}^{-}$, pH 6.5) at concentrations that are well tolerated in humans. Here, we used a transposon mutagenesis approach to identify *PA* mutants that are hypersensitive to ${\text{A-NO}}_{2}^{-}$. Among greater than 10,000 mutants screened, we focused on *PA4455*, in which the transposon was found to disrupt the production of a putative cytoplasmic membrane-spanning ABC transporter permease. The *PA4455* mutant was not only highly sensitive to ${\text{A-NO}}_{2}^{-}$, but also the membrane perturbing agent, EDTA and the antibiotics doxycycline, tigecycline, colistin, and chloramphenicol, respectively. Treatment of bacteria with ${\text{A-NO}}_{2}^{-}$ plus EDTA, however, had the most dramatic and synergistic effect, with virtually all bacteria killed by 10 mM ${\text{A-NO}}_{2}^{-}$, and EDTA (1 mM, aerobic, anaerobic). Most importantly, the *PA4455* mutant was also sensitive to ${\text{A-NO}}_{2}^{-}$ in biofilms. ${\text{A-NO}}_{2}^{-}$ sensitivity and an anaerobic growth defect was also noted in two mutants (*rmlC* and *wbpM*) that are defective in B-band LPS synthesis, potentially indicating a membrane defect in the *PA4455* mutant. Finally, this study describes a gene, *PA4455*, that when mutated, allows for dramatic sensitivity to the potential therapeutic agent, ${\text{A-NO}}_{2}^{-}$ as well as EDTA. Furthermore, the synergy between the two compounds could offer future benefits against antibiotic resistant *PA* strains.

## Introduction

A hallmark of two major airway diseases, chronic obstructive pulmonary disease (COPD) and cystic fibrosis (CF), is infection by the opportunistic Gram-negative, rod-shaped pathogen, *Pseudomonas aeruginosa (PA)*. COPD is characterized by airway epithelial debridement, the primary cause of which is chronic smoking (Hassett et al., 2014). In contrast, CF is an autosomal recessive inherited disease resulting in mutations in the cystic fibrosis transmembrane regulator gene (CFTR) on chromosome 7 that affects the function of many organs including but not limited to the lung, liver, pancreas, intestine, skin, and testes (Boucher, 2002). However, with time, it is airway infection in CF that leads to pulmonary insufficiency and ultimately death of the patient at an average age of ~39 (MacKenzie et al., 2014). Complicating both diseases are *in vivo* mutations that occur in the *mucA* gene of *PA*, encoding an inner membrane spanning anti-sigma factor (Martin et al., 1993; Valderrey et al., 2010). The most common mutation, *mucA22*, is characterized by a deletion of a G residue at base 430, resulting in a truncated MucA protein of ~15.8 kDa (Martin et al., 1993); wild-type MucA is 21 kDa. Most notably, *mucA* mutations allow for the sigma factor AlgT(U) (alternatively σ^E^ or σ^22^) to transcribe genes involved in the regulation and production of a highly viscous exopolysaccharide known as alginate. The production of alginate allows for increased resistance to antibiotics (Hentzer et al., 2001; Murphy et al., 2008), phagocytic killing (Meshulam et al., 1984), and markedly decreases the overall pulmonary function in both COPD and CF patients, especially during exacerbations of both diseases (Govan and Deretic, 1996; Hassett et al., 2014). Often, multiple antibiotic classes including aminoglycosides, β-lactams, quinolones, macrolides, and polymyxins are used to combat such refractory infections, yet during chronic infections, frequent use of these antibiotics results in strains that develop resistance. Therefore, novel treatment strategies are needed to reduce or even completely eradicate such problematic organisms.

In 2006, our group reported an important and novel observation that slightly acidified nitrite (${\text{A-NO}}_{2}^{-}$) at the pH resembling that of the CF airway surface liquid (6.3–6.5; Coakley et al., 2003; Yoon et al., 2006) killed mucoid *PA* in both planktonic and biofilm culture, in sterile CF airway transplant sputum, and the findings were also substantiated in a murine lung infection model (Yoon et al., 2006). ${\text{A-NO}}_{2}^{-}$ displayed no adverse effects on primary CF airway epithelia when used at levels 20-fold higher (300 mM) than that required to kill mucoid *PA* (Yoon et al., 2006). Recent studies on *PA* biofilms grown on CF primary epithelial cells showed that ${\text{A-NO}}_{2}^{-}$ killed *PA* biofilms, inhibited the activity of aminoglycosides, yet demonstrated synergy with colistomethate (Zemke et al., 2014, 2015). Toxicology studies using dogs and rats revealed that inhalation of nebulized NaNO_2_ for 28 days was well tolerated in rats and dogs at doses up to 19–20 mg/kg: doses where the no observed adverse effect level (NOAEL) was established (Major et al., 2010). In addition, from a human use standpoint, ${\text{NO}}_{2}^{-}$ is already used as a food preservative of red meats and a treatment for cyanide poisoning.

In this study, we first used a transposon (Tn) mutant approach to screen for PA mutants that demonstrate increased susceptibility to ${\text{A-NO}}_{2}^{-}$ when compared to wild-type and a mucoid *mucA22* mutant. From a panel of ~10,000 Tn mutants, we focused on a very sensitive mutant that had a Tn insertion in the *PA4455* gene, encoding a putative cytoplasmic membrane-spanning ABC transporter permease. ATP-binding cassette transporters (or ABC transporters) are members of a protein superfamily that are present throughout various phyla, ranging from bacteria to humans (Higgins, 1992). These proteins utilize ATP to import small molecules including nutrients, antibiotics, drugs, and a myriad of other molecules. The *PA4455* mutant was extremely sensitive to ${\text{A-NO}}_{2}^{-}$, EDTA and several antibiotics. We also found that two mutants unable to generate the B-band lipopolysaccharide/O-side chain, *rmlC* and *wbpM*, are defective in anaerobic growth and demonstrate increased susceptibility to both ${\text{A-NO}}_{2}^{-}$ and EDTA, and especially when the two reagents were combined. From a translational perspective, a means to target PA4455 with novel drugs could possibly enhance the overall efficiency of killing problematic *PA* infections, especially those associated with CF and COPD.

## Materials and methods

### Bacterial strains and growth conditions

The bacteria and all primers designed and used for PCR reactions used in this study are listed in Table 1. Organisms were grown in either Luria-Bertani broth (LB), LB-100 mM KNO_3_ (LBN) ± 50 mM potassium phosphate, pH 6.5 or *Pseudomonas* isolation agar (PIA). Aerobic cultures were grown at 37°C with shaking at 180 rpm at a 1/10 volume to total Erlenmeyer flask volume. Anaerobic cultures were grown in a Coy laboratories anaerobic chamber at 37°C. Media were also solidified with 1.5% Bacto-agar. Frozen bacterial stocks were stored at −80°C in a 1:1 mixture of 30% glycerol and stationary-phase bacterial suspension.

**Table 1:** **Bacterial strains, plasmids and oligonucleotides used in this study**.

**Strain, plasmid or oligonucleotide**	**Description (relevant genotype or phenotype) or sequence (5′ to 3′)**	**Source, reference, or RE site**
***E. coli***		
DH5α	F^−^Φ80d*lacZΔM15 endA1 recA1 hsdR17(r${}_{K}^{-}$ m${}_{K}^{-}$) supE44 thi-1 gyrA96* Δ*(lacZYA-argF)*U169	Invitrogen
S17-1 λ *pir*	Pro- Res- Mod+ *recA*; integrated RP4-Tet::Mu-Kan::Tn*7*, Mob+	Simon et al., 1983
***P. aeruginosa***		
PAO1	Wild-type, prototroph	Holloway et al., 1979
PAO1Δ*mucA*	PAO1 deletion mutant of *mucA* gene	This study
*PA4455*::Tn-Gm^r^	PAO1 transposon mutant	This study
*PA4455*::Gm	PAO1Δ*PA4455*::Gm insertion mutant	This study
*rmlC*	PAO1 derivative, *rmlC*::Gm insertion, A^−^B^−^	Rahim et al., 2000
*rmd* R1 _05_, R2 _O5_	PAO1 derivative, *rmd*::Gm insertion, A^−^B^+^	Rocchetta et al., 1998a,b
*wbpM*	PAO1 derivative, *wbpM*::Gm insertion, A^+^B^−^	Creuzenet and Lam, 2001
*PA4455*::Gm (pUCP-*PA4455*)	Complemented *PA4455*::Gm mutant by plasmid-borne pUCP-*PA4455*	This study
PAO1::*attB*::*PA4455*-*lacZ*	Promoter of *PA4455* and *lacZ* transcriptional fusion was integrated at the chromosomal *attB* site of PAO1	This study
**PLASMIDS**		
pUCGM	Source for Gm^r^ cassette, Ap^R^, Gm^R^	Schweizer, 1993
pQF50	Broad-host-range transcriptional fusion vector with a promoterless *lacZ*, Ap^R^	Farinha and Helinski, 1990
mini-CTX1	Integration proficient plasmid for *P. aeruginosa*, Tc^R^	Hoang et al., 2000
pEX100T-KS	*Pseudomonas* gene replacement suicide vector with modified multiple cloning site*, sacB, oriT*, Cb^R^	Schweizer and Hoang, 1995
pUCPPA4455	798 bp PA4455 gene cloned into *Eco*RI/*Bam*HI sites of pUCP20, Ap^R^	This study
pHA531	*nirS::lacZ* transcriptional fusion in pQF50, Ap^R^	Arai et al., 1995
pHA532	*nirQ::lacZ* transcriptional fusion in pQF50, Ap^R^	Arai et al., 1995
pHA533	*norC::lacZ* transcriptional fusion in pQF50, Ap^R^	Arai et al., 1995
pQF50-*narK*	*narK* promoter cloned into pQF50, Ap^R^	This study
mini-CTX1-PA4455-*lacZ*	A 4-kb fragment containing promoter of *PA4455* and *lacZ* fusion cloned into *Xho*I/HindIII sites of the vector pmini-CTX1, Tc^R^	This study
pEXΔPA4455::Gm	A 4-kb fragment containing flanking sequences of *PA4455* and transposon insertion cassette cloned into *Not*I/*Spe*I sites of pEX100T-KS, Cb^R^, Gm^R^	This study
pEXΔOPABC::FRT-Gm	A 3-kb fragment containing flanking sequences of the operon from *PA4452* to *PA4456* and the FRT-Gm^R^-FRT cassette cloned into *Eco*RI/*Bam*HI sites of pEX100T-KS, Cb^R^, Gm^R^	This study
**OLIGONUCLEOTIDES**		
AD2	5′-CANGCTWSGTNTSCAA	Caetano-Anolles, 1993
GM447	5′-GTGCAAGCAGATTACGGTGACGAT	Caetano-Anolles, 1993
GM464	5′-TGACGATCCCGCAGTGGCTCTC	Caetano-Anolles, 1993
Pr4455/Xh5′	CCGCTCGAGCGAATGTGGCGCCTGGAT	This study
Pr4455/Bm3′	CGGGATCCCATCAGGACTCCTTGCGATGC	This study
PA4455/Eco5′	GGAATTCATGACTGGGAGAGCGCTGATGCGTAGAGTCTCTCCCCTC	This study
PA4455/Bm3′	CGGGATCCTCAGAAATCTCCAAACATCAAAGC	This study
**qPCR OLIGONUCLEOTIDES**		
PA4452 FWD	CCTGTTGCTGGCCTTCATA	This study
PA4452 REV	CTGCTGACCTTGGCAATCT	This study
PA4453 FWD	AACGCGCTGCTCGAATA	This study
PA4453 REV	CTGTCACGGATCTCCATGTT	This study
PA4454 FWD	GACCATGGAGATCAACCAGAAC	This study
PA4454 REV	GACGCTGATGCCGATGTATT	This study
PA4455 FWD	ATGAAGGCCACCGAACAG	This study
PA4455 REV	CAGCGGCATGGAGATGAA	This study
PA4456 FWD	TCACCGATCTCGACGTCTT	This study
PA4456 REV	GAAGCTTCATCAGGACGATGT	This study
*acpP* FWD	AGGAAGAAGAAGTCACCAACAG	This study
*acpP* REV	TCCTCTTCCAGAGCCATCA	This study

*Ap^R^, ampicillin resistant; Cb^R^, carbenicillin resistant; Tc^R^, tetracycline resistant; Gm^R^, gentamycin resistant*.

### Manipulation of recombinant DNA and genetic techniques

All plasmid and chromosomal nucleic acid manipulations were performed by standard techniques (Maniatis et al., 1982). Plasmid DNA was transformed into *E. coli* strain DH5α-MCR (Protein Express, Cincinnati, OH). To detect the presence of insert DNA, X-Gal (5-bromo-4-chloro-3-indolyl-β-D-galactopyranoside; 40 μg/ml) was added to agar media. Restriction endonucleases and T4 DNA ligase were used as specified by the vendor (Invitrogen/Gibco-BRL Corp., Gaithersburg, MD). Plasmid DNA was isolated using the QIAprep Spin Miniprep Kit (Qiagen) and restriction fragments were recovered from low melting point agarose gels. PCRs for genes of interest were performed by using Pfu DNA polymerase (BRL) and appropriate primers in an MJ Research thermal cycler, with 30 cycles of denaturation (2 min, 94°C), annealing (1 min, 54°C), and extension (1 min 30 s, 72°C). Amplified DNA fragments were gel purified, cloned into pCR2.1 (Invitrogen), and sequenced.

### Construction of *PA* mutants

#### Transposon (Tn) mutagenesis

Wild-type strain PAO1 was subjected to transposon mutagenesis using the mariner transposon vector, pBT20 (Kulasekara et al., 2005). The transposon within pBT20 was conjugally transferred by biparental mating using *E. coli* S17-1 λ *pir* into strain PAO1 following a protocol described elsewhere (Kulasekara et al., 2005). Mating mixtures were scraped and resuspended in 1 ml of L-broth. Suspensions (300 μl) were spread evenly onto *Pseudomonas* isolation agar (PIA) plates containing gentamicin (Gm) at 150 μg/ml, and incubated at 37°C for 48 h. Conjugants were picked and patched onto freshly prepared LBN plates, pH 6.5 containing 15 mM ${\text{A-NO}}_{2}^{-}$ and further incubated again at 37°C for 48 h. ${\text{A-NO}}_{2}^{-}$ sensitive conjugants were selected, purified and confirmed for susceptibility to ${\text{A-NO}}_{2}^{-}$. Transposon insertion sites were determined through sequencing the flanking region of the transposon by a semi-random PCR method, as described previously (Caetano-Anolles, 1993) using random primer AD2 and transposon-specific primer Gm447, followed by the nested primers Gm464 and AD2 (Table 1).

#### Allelic exchange, sucrose counter-selection

The strategy for insertional inactivation of some of the genes, primarily *PA4455* derivatives listed in Table 1 was facilitated by gene disruption with an 850-bp gentamicin resistance (Gm^R^) cassette from pUCGM (Schweizer, 1993). The gene replacement vector pEX100T (Schweizer, 1992) was used to allow for selection of double-crossover events within putative recombinants cultured on LB-agar medium containing 150 μg/ml Gm and subsequently with 7% sucrose for counterselection. All mutants were confirmed by PCR and DNA sequencinxg analysis.

### qPCR analysis of wild-type and mutant strains

Strains PAO1, *PA4455*, and *PA4455* complemented with pUCP-PA4455 were grown overnight in LB media. The strains were then sub-cultured the next day into fresh LB and allowed to grow to late log phase. The bacteria were then harvested and subjected to RNA extraction using an RNeasy Protect Bacteria Mini Kit (Qiagen), including a DNase reaction to remove endogenous genomic DNA. Equal amounts of RNA were used in a Reverse Transcriptase Reaction (Im-Prom II Reverse Transcription System) to obtain total cDNA that was then used in a PCR reaction to confirm qualitatively the absence of the gene from the mutant strain. Subsequently, the same samples were used in a qPCR reaction using the StepOnePlus System (Thermo Scientific, Applied Biosystems). Amplification was detected using PowerUp SYBR Green Master Mix (Applied Biosystems). Primers directed against *PA4452, PA4453, PA4454, PA4455, PA4456* and a “housekeeping” gene, *acpP*, were developed using IDT primer design tools (Integrated DNA Technologies) to create ~100-bp fragments, so as to minimize errors between gene amplification measurements. Threshold cycle values were used to determine relative gene expression using the Δ*ΔC*_T_ method (Livak and Schmittgen, 2001).

### SDS, EDTA, and NaNO_2_ killing studies

Aerobically grown bacteria were serially diluted on square Petri dishes containing either LB or LBN agar, pH 6.5, ± 7.5 or 17 mM ${\text{A-NO}}_{2}^{-}$, 0.5% SDS, or 1 mM EDTA. Details of the strains used for these studies are provided in the figure legends. The diluted suspensions were spotted (10 μl) on the surfaces of the plates and the organisms allowed to incubate at 37°C under aerobic or anaerobic conditions, the latter using a Coy Laboratory Anaerobic Chamber (Grass Lake, MI).

### P_*PA*4455_–*lacZ* fusion and complementary plasmid construction

The P_*PA*4455_−*lacZ* transcriptional fusion was constructed by amplifying the *PA4455* operon promoter region, encompassing nucleotides 810–1223 bp upstream of the start codon of *PA4455*. PCR was performed using primers Pr4455/Xh5′ and Pr4455/Bm3′ with PAO1 chromosomal DNA. The resulting 413-bp fragment was excised with XhoI and BamHI, and ligated to a 3-kb BamHI/HindIII-digested lacZ fragment. The resultant fusion was then cloned between the XhoI and HindIII sites of mini-CTX1 to yield mini-CTX1-P_*PA*4455_−*lacZ*. Chromosomal integration of this mini-CTX1-P_*PA*4455_−*lacZ* fusion vector in *PA*, excision of unwanted plasmid sequences and verification of insertion at the chromosomal *attB* locus were performed as described previously (Hoang et al., 2000). For genetic complementation studies, the coding region of *PA4455* was amplified from PAO1 chromosomal DNA using oligonucleotides PA4455/Eco5′ and PA4455/Bm3′. The EcoRI/BamHI-digested PCR product was cloned into pUCP20 (Schweizer, 1991) that had been digested with the same enzymes. Competent cells of the PAO1 *PA4455* mutant were prepared and electroporated with 50 ng of the plasmid, pUC-*PA4455*. Transformants were selected on PIA plates containing carbenicillin at 500 μg/ml.

### *lacZ* transcriptional analysis of P_*PA*4455_-*lacZ* and pQF50 derivative containing strains

For P_*PA*4455_−*lacZ*, overnight cultures of PAO1 attB::P_*PA*4455_−*lacZ* were diluted 1/100 into 50 ml of fresh LBN pH 6.5 and grown to early log phase, after which the samples were aliquoted into test tubes containing the respective treatments. These were then incubated for 2 h aerobically with shaking or 2.5 h anaerobically, both at 37°C. For pQF50 derivative strains, overnight aerobic cultures of the bacteria were inoculated 1/100 into fresh LBN, and the cultures were incubated aerobically or anaerobically overnight. Analysis of the activation of *lacZ* was carried out using methods described by Miller using slight modifications (Miller, 1992; Su et al., 2014). Briefly, a 400-μl suspension of bacteria (OD600 ~1.0) was transferred to a glass tube and mixed with 600 μl of Z-buffer. Twenty μl of 0.1% SDS and 40 μl of chloroform were then added to the samples and vortexed. The samples were then incubated in a 28°C water bath for 5 min, after which 200 μl of an ortho-nitrophenyl-β-galactoside solution (ONPG, 4 mg/ml) was added to each sample. After briefly vortexing a timer was started and the samples were returned to the 28°C water bath. Reactions were stopped by the addition of 500 μl of a 1 M sodium carbonate solution. The samples were then centrifuged and 1 ml of the supernatant was analyzed spectrophotometrically at 420 nm. Utilizing the OD_600_, reaction time, and A_420_, the Miller units of each sample were then calculated. Results were analyzed using SPSS software (IBM, version 23). For pQF50 variants, independent *T*-tests were used to determine significance between PAO1 and *PA4455* for each plasmid. For P_*PA*4455_−*lacZ* assays, one-way ANOVA was utilized to determine the significant differences among the group.

### Confocal laser scanning microscopic analysis for determination of ${\text{A-NO}}_{2}^{-}$ sensitivity of *PA* biofilms

Biofilms were grown in glass-bottomed culture dishes (MatTek, Ashland, MA) essentially as described by Yoon et al. (2002). Briefly, overnight cultures were grown in L-broth to stationary phase and diluted 1:100 into the culture dishes containing 3 ml of LBN. Samples were incubated anaerobically at 37°C for 24 h. Planktonic cells were removed by washing the wells twice with PBS and fresh L-broth-50 mM potassium phosphate, pH 6.5 containing 15 mM KNO_3_ (control) or 15 mM KNO_3_ plus 15 mM NaNO_2_. Cultures were incubated in an anaerobic chamber for 2 days at 37°C. After removing the planktonic cells by two washes with PBS, biofilm bacteria were exposed to a cell viability stain (BacLight, Invitrogen) composed of SYTO 9 and propidium iodine. Biofilm images were obtained using an LSM 710 confocal microscope (Zeiss, Heidelberg, Germany). The excitation and emission wavelengths for green fluorescence were 488 and 500 nm, while those for red fluorescence were 490 and 635 nm, respectively. All biofilm experiments were repeated indepently at least 3 times. The live/dead ratios of the biofilms were calculated using the ImageJ software. The percentage of dead/live ratio was normalized after comparisons between treated (${\text{NO}}_{3}^{-}$ plus ${\text{NO}}_{2}^{-}$) and control (${\text{NO}}_{3}^{-}$ alone).

### Antibiotic MIC determinations

Minimum inhibitory concentration (MIC) testing was performed using the Etest system (bioMerieux, Marcy-l-Etoile, France) following manufacturer guidelines. Testing was performed using 150 mm cation adjusted Mueller Hinton agar plates (BD Diagnostics, Sparks, MD) incubated at 35°C for 18–24 h in ambient air.

### Mouse acute pneumonia infection model

Six-week old male BALB/c mice (19–21 g) were purchased from Taconics (Cambridge City, IN). All animals were housed in positively ventilated microisolator cages with automatic recirculating water located in a room with laminar, high efficiency particulate-filtered air. The animals received autoclaved food, water, and bedding. Mice were handled in accordance with approved protocols through the Institutional Animal Care and Use Committee at the University of Illinois at Urbana-Champaign. Mice were infected intranasally with 1 × 10^6^ CFU of strain PAO1, the *PA4455* mutant, or the complemented strain (pUCP-*PA4455*; Zhang et al., 2005; Hao et al., 2011). After 20 h, mice were euthanized and lungs were harvested, homogenized, serially diluted and plated on PIA with or without 400 μg/ml carbenicillin for determination of bacterial load.

### Transmission electron microscopy (TEM) of bacterial samples

Overnight cultures were diluted 1:100 in fresh L-broth and incubated at 37°C with shaking until late log phase. Culture samples were removed and adjusted to an OD_600_ of 1.0 and the bacteria clarified by centrifugation at 2600 RPM (~600 × ^g^) for 5 min. The cells were then prepared for TEM using a protocol described by Kim et al. (2007) with slight modifications. The cells were washed three times in 0.1 M sodium cacodylate, then stained and fixed with a 1:1 mixture of 1% osmium tetraoxide with 7.5 mg/ml of potassium ferricyanide and a 5% glutaraldehyde solution. After 1 h, the cells were washed again with 0.1 M sodium cacodylate and subjected to a graded series of ethanol (25–100%), each for 15 min, followed by a 100% acetone wash for another 15 min. The cells were infiltrated and embedded in LX-112 Resin (Ladd Research Industries). The resin was sectioned using a Leica EM UC 7 ultramicrotome to 100 nm. Images were taken on a Hitachi H 750 transmission electron microscope operating at 100 kV.

## Results

### Identification of *PA4455*, encoding a putative ABC transporter permease: a gene critical for resistance to ${\text{A-NO}}_{2}^{-}$

**Figure 1:**
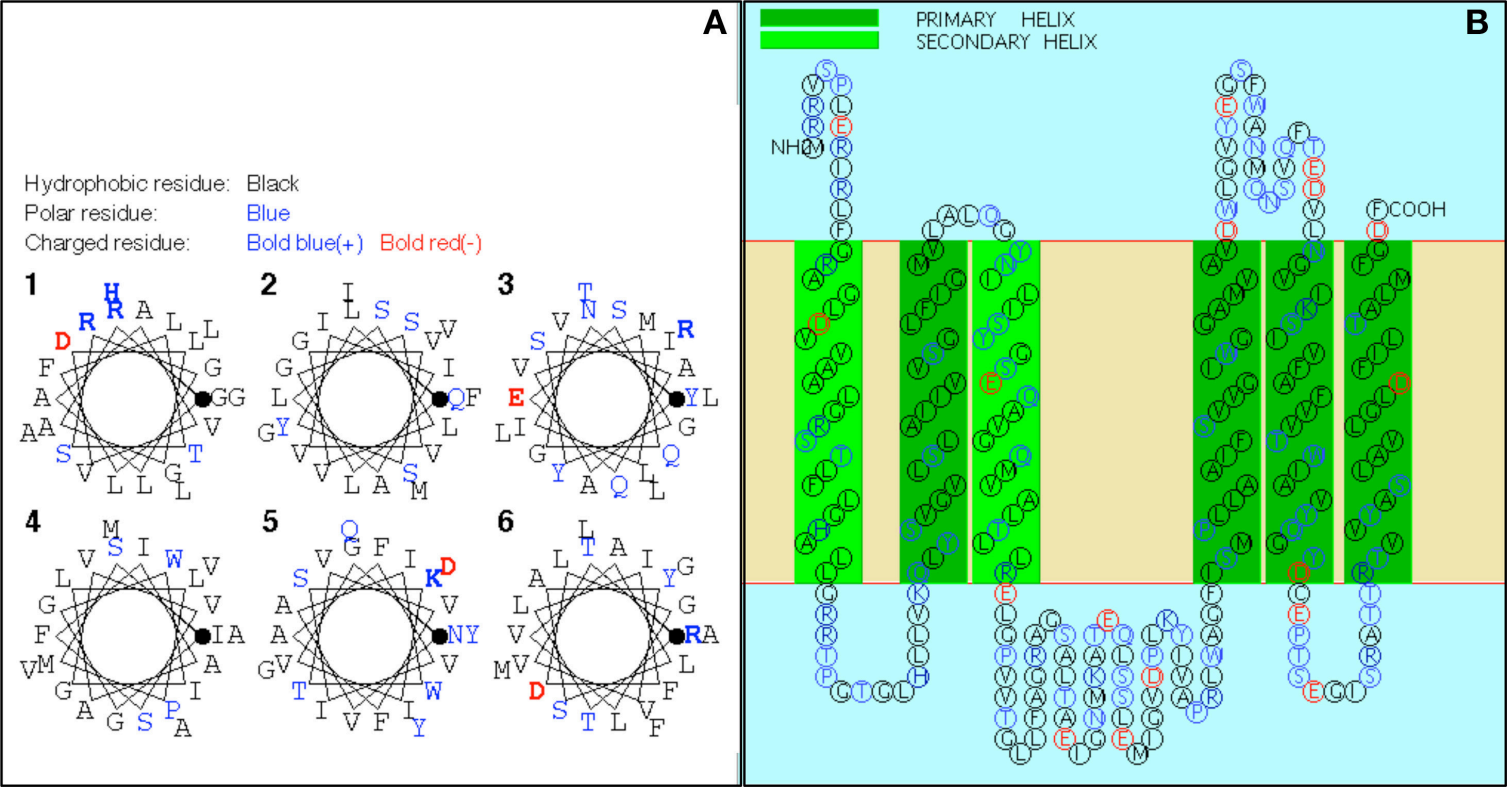
**Software predictions of the transmembrane domains of PA4455**. **(A)** Helical wheel diagram of predicted transmembrane segments of PA4455. Hydrophobic residues, black; Polar residues, blue; Charged residues, Bold blue (+) and Bold red (−). **(B)** Predicted membrane topology of PA4455 within the cytoplasmic membrane. Primary and secondary membrane spanning helices are indicated by dark or light green color, respectively.

In this study, we used a Tn mutagenesis approach to identify mutants that demonstrated increased sensitivity to ${\text{A-NO}}_{2}^{-}$ relative to wild-type bacteria. Colonies that were patched from PIA-Gm plates onto PIA-Gm/${\text{A-NO}}_{2}^{-}$ plates that showed little or no growth were screened for ${\text{A-NO}}_{2}^{-}$ sensitivity followed by sequencing of the Tn-interrupted gene. The most sensitive mutant whose gene product was not deemed cytoplasmic in location based upon the membrane topology program SOSUI (http://harrier.nagahama-i-bio.ac.jp/sosui/sosui_submit.html), and hence a potential viable drug target, was that involving a Tn insertion within the *PA4455* gene. The *PA4455* gene encodes a putative ABC transporter permease that has 6 predicted membrane-spanning domains (Figures 1A,B) within the cytoplasmic membrane (Class 3). However, experimental evidence also shows that the 28.4-kDa protein encoded by *PA4455* can also be localized within membrane vesicles (class I) based upon MALDI-TOF mass spectrometric analysis (www.pseudomonas.com; Choi et al., 2011). This predicted topology was also supported by other cellular localization programs including TOPPRED (https://bioweb.pasteur.fr/packages/pack@toppred@1.10) and PSORTb V. 3.0 (http://www.psort.org/psortb/; data not shown).

**Figure 2:**
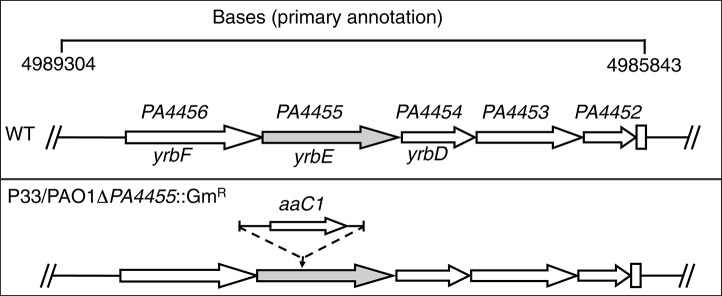
**Schematic diagram of the strategy to generate a deletion mutant of the ***PA4455*** gene**. The genomic region of *PA* spanning base pairs 4989304–4985843 are shown at the top of the figure. The designated genes *yrbF, yrbE*, and *yrbD* are based upon homology to *Bacillus subtilis* genes as analyzed by www.pseudomonas.com. The second construct, PAO1 *PA4455*::Gm^R^, harbors a gentamicin resistance cassette (Gm^R^) interrupting the *PA4455* gene.

The initial transponson mutant (designated P33) had a Tn insertion located 436 bp from the 5′ end of the *PA4455* ORF with Gm resistance determinant (*aaC1*) transcribed in the same orientation as *PA4455* (Figure 2). We believed it was unlikely that the Tn insertion would exert any polar effects on downstream genes that included *PA4454, PA4453*, and *PA4452*, each of which encode conserved hypothetical proteins, respectively. We next constructed a *PA4455* knockout mutant using the standard strategy of insertional disruption with a gentamycin cassette (Gm^R^) followed by allelic exchange (Figure 2). The resultant mutant strain, PAO1 *PA4455*::Gm^R^, exhibited the same level of sensitivity to ${\text{A-NO}}_{2}^{-}$ as the Tn mutant P33. We did not believe this mutant would have any polar effects on nearby genes. Although modest increases in mRNA levels of up- and down-stream genes were observed in the mutants, we do not believe this is causing the phenotypic effects observed in this work because of the complementation of each phenotype by provision of *PA4455 in trans*. This was confirmed by transcriptional analysis of the aforementioned genes using qPCR, comparing the gene expression to a previously used housekeeping gene *acpP* (Supplementary Figure 1) from both aerobically and anaerobically grown samples (Lenz et al., 2008). Both mutant strains displayed no growth defect in regular LB or LBN media compared to the wild-type strain PAO1 (data not shown). Thus, we used PAO1-*PA4455*::Gm^R^ (herein *PA4455* mutant) for the remainder of our studies.

### Sensitivity of planktonic *PA4455* mutant bacteria to ${\text{A-NO}}_{2}^{-}$ under aerobic and anaerobic conditions

**Figure 3:**
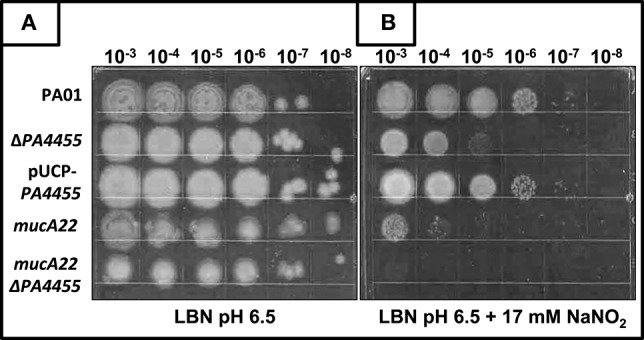
**Sensitivity of planktonic ***PA4455*** mutant bacteria to ${\text{A-NO}}_{2}^{-}$ under aerobic conditions**. Serial dilutions (x-axis) of aerobically grown stationary phase bacteria were spotted onto LBN plates **(A**, control) or **(B)** LBN plates ± 17 mM ${\text{A-NO}}_{2}^{-}$ and incubated aerobically for 48 h at 37°C. The lids were removed and the plates were scanned by an HP Scanjet 7400c using Adobe Photoshop and edited for visual clarity using Powerpoint:Mac2011 software.

**Figure 4:**
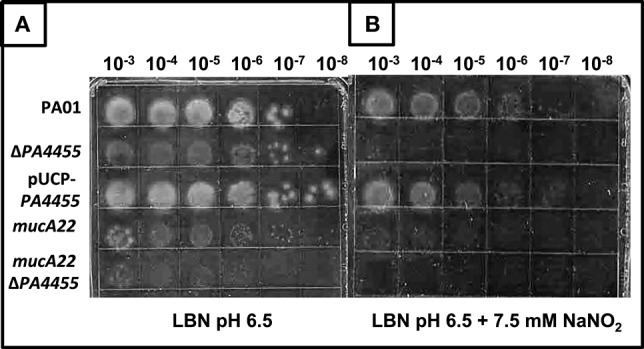
**Sensitivity of planktonic ***PA4455*** mutant bacteria to ${\text{A-NO}}_{2}^{-}$ under anaerobic conditions**. Serial dilutions (x-axis) of aerobically grown stationary phase bacteria were spotted onto LBN plates (**A**, control) or **(B)** ± 7.5 mM ${\text{A-NO}}_{2}^{-}$ and incubated anaerobically for 48–72 h at 37°C. The lids were removed and the plates were scanned by an HP Scanjet 7400c using Adobe Photoshop and edited for visual clarity using Powerpoint:Mac2011 software.

Using a simple serial dilution plating assay, we monitored the sensitivity of the *PA4455* mutant bacteria relative to wild-type bacteria and complemented strains. Serially diluted bacterial suspensions were spotted onto LBN plates ± 17 mM ${\text{A-NO}}_{2}^{-}$ (aerobic) or 7.5 mM ${\text{A-NO}}_{2}^{-}$ (anaerobic), respectively, based on preliminary experiments which determined these concentrations as a minimum to discern a noticeable effect on viability (data not shown). The results in Figure 3B (compared to Figure 3A controls) demonstrates that the *PA4455* mutant is dramatically more sensitive to ${\text{A-NO}}_{2}^{-}$ than wild-type bacteria under both aerobic and, most dramatically, under anaerobic conditions (Figure 4B compared to Figure 4A). Complementation was achieved in trans on a multi-copy plasmid, pUCP-*PA4455*. For comparison, we also used mucoid *mucA22* (previously shown to be sensitive to ${\text{A-NO}}_{2}^{-}$ Yoon et al., 2006) and a *mucA22 PA4455* double mutant. Interestingly, both *mucA22* and the double mutant bacteria were indistinguishable in anaerobic ${\text{A-NO}}_{2}^{-}$ sensitivity relative to the *PA4455* mutant alone (Figure 4B) yet the double mutant was ~10-fold more sensitive to ${\text{A-NO}}_{2}^{-}$ under aerobic conditions (Figure 3B).

### Sensitivity of biofilm *PA4455* mutant bacteria to ${\text{A-NO}}_{2}^{-}$ under anaerobic conditions

**Figure 5:**
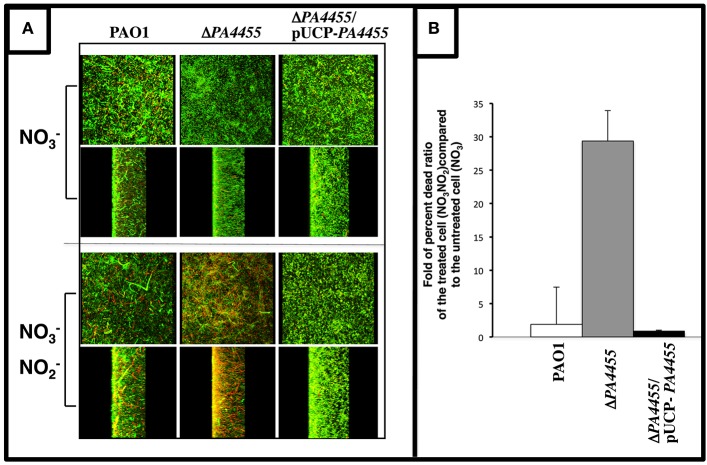
**Sensitivity of biofilm ***PA4455*** mutant bacteria to ${\text{A-NO}}_{2}^{-}$ under anaerobic conditions. (A)** Biofilms were grown anaerobically in LBN for 24 h in glass bottomed culture dishes. Spent medium was gently aspirated and replaced with LBN-50 mM potassium phosphate, pH 6.5, containing 15 mM NaNO_2_. Cultures were incubated in an anaerobic chamber for 2 days at 37°C. Biofilms were then stained a cell viability stain (*Bac*Light, InVitrogen) composed of SYTO 9 and propidium iodine. Biofilm images were obtained using an LSM 510 confocal microscope (Carl Zeiss, Inc., Germany). The excitation and emission wavelengths for green fluorescence were 488 nm and 500 nm, while those for red fluorescence were 490 nm and 635 nm, respectively. All biofilm experiments were repeated at least 3 times. **(B)** The live/dead ratios of the biofilms were calculated using ImageJ image processing program.

Since ${\text{A-NO}}_{2}^{-}$ sensitivity in *PA* is more pronounced under anaerobic conditions (Yoon et al., 2006), we next elected to grow bacteria as a biofilm under anaerobic conditions via ${\text{NO}}_{3}^{-}$ respiration and assess the relative sensitivity of wild-type and the *PA4455* mutant to ${\text{A-NO}}_{2}^{-}$. We have previously shown that anaerobic conditions favor more robust biofilm formation than under aerobic conditions (Yoon et al., 2002). In this experiment, we observed that the *PA4455* mutant is highly sensitive to ${\text{A-NO}}_{2}^{-}$ when compared to wild-type and complemented bacteria, as shown by a shift in the live/dead ratio of the biofilm bacteria (Figures 5A,B).

### Denitrification pathway transcriptional activities are increased under anaerobic conditions in the *PA4455* mutant relative to wild-type bacteria

**Figure 6:**
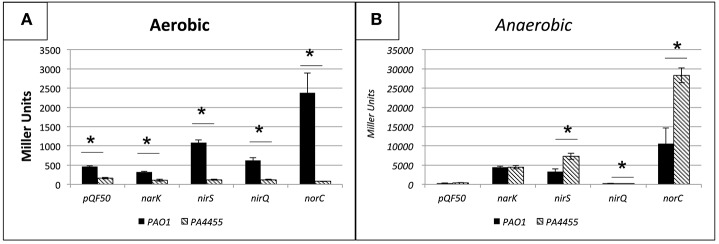
**Transcriptional activity of the ***nirS, nirQ, norC***, and ***narK*** promoters probed by ***lacZ*** fusion analysis under aerobic (A) versus anaerobic (B) conditions**. *PA* PAO1 (wild-type) and its isogenic Δ*PA4455*::Gm^R^ mutant were transformed with pQF50 (vector control), pHA531 (*nirS::lacZ*), pHA532 (*nirQ::lacZ*), pHA533 (*norC::lacZ*), or pQF-*narK* (*narK::lacZ*), respectively. β-galactosidase activity was measured in cells grown in LBN broth under aerobic and anaerobic conditions. Reported values are the averages of 4 independent experiments. (^*^) designates >95% confidence.

To evaluate whether transcription of genes involved in anaerobic respiration were potentially altered in the *PA4455* mutant relative to wild-type bacteria that may alter the overall sensitivity to ${\text{A-NO}}_{2}^{-}$ of the *PA4455* mutant observed in Figures 3B, 4B, we assessed the aerobic and anaerobic transcriptional activity of the *nirS* (encoding nitrite reductase), *nirQ* (encoding the NirQ anaerobic regulator), *norC* (encoding the NorC component of NO reductase) and *narK* (encoding the NarK efflux pump) promoters using classical *lacZ* fusion analyses. *PA* strains PAO1 (wild-type) and its isogenic *PA4455* mutant were transformed with pQF50 (vector control), pHA531 *(nirS::lacZ)*, pHA532 *(nirQ::lacZ)*, pHA533 *(norC::lacZ)*, or pQF-narK *(narK::lacZ)*, respectively. Next, β-galactosidase activity was measured in cells grown in LBN broth under aerobic and anaerobic conditions overnight. In support of our hypothesis, the *PA4455* mutant exhibited a clear upregulation of several of the aforementioned genes anaerobically, indicating that ${\text{NO}}_{3}^{-}$ may more readily pass through the membrane of the mutant and trigger transcriptional activation of these genes. This presumably could apply to ${\text{NO}}_{2}^{-}$ as well, explaining the increased sensitivity to it. Under aerobic conditions, transcriptional activity was noticeably lower in strain *PA4455* when compared to wild-type levels, which, although unexpected, indicate that perhaps the cell has developed an altered preference for the available nitrogen sources (Figure 6A). Anaerobically, when the denitrification pathway is active, gene activation levels increased dramatically (often ~2-fold higher) in the *PA4455* mutant relative to the wild-type in *nirS*, and *norC* transcription, but interestingly not in *narK* transcription (Figure 6B). Unexpectedly, *nirQ* was lower anaerobically in *PA4455* than in PAO1, although both levels were also lower than their respective controls.

### Response of the *PA4455* mutant to membrane stressors: sensitivity to the chelator EDTA but not SDS

**Figure 7:**
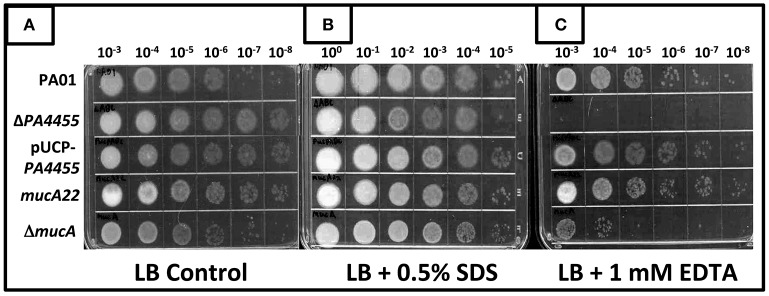
**Sensitivity of various mutant strains to the membrane permeabilizing agents SDS and EDTA**. Serial dilutions (x-axis) of aerobically grown stationary phase bacteria were spotted onto LBN plates **(A)**, control, **(B)** 0.5% SDS, or **(C)** 1 mM EDTA and incubated aerobically for 48 h at 37°C. The lids were removed and the plates were scanned by an HP Scanjet 7400c using Adobe Photoshop and edited for visual clarity using Powerpoint:Mac2011 software.

SDS has been shown more than 40 years ago to cause significant bacterial membrane damage with subsequent release of DNA, RNA and protein (Woldringh and van Iterson, 1972). It has also long been known that the chelator EDTA has pleotropic effects on the lipopolysaccharide of *PA* (Champlin et al., 2005; Alakomi et al., 2006). Thus, we postulated that the *PA4455* mutant, because of an absence of the 6 membrane-spanning protein, *PA4455*, may be unusually sensitive to not only ${\text{A-NO}}_{2}^{-}$ (Figures 3–5) but also to EDTA and SDS. In addition, its homolog in *E. coli* demonstrated sensitivity to EDTA and several other membrane stressors, suggesting that perhaps this phenomenon would exist in *PA* as well (Malinverni and Silhavy, 2009). The results displayed in Figure 7B clearly indicate that the wild-type and all tested mutant and complemented bacteria were not sensitive to 0.5% SDS relative to control bacteria (Figure 7A). In contrast, treatment of bacteria with 1 mM EDTA clearly killed the vast majority of the *PA4455* mutant bacteria (Figure 7C). We also tested alginate-overproducing strains *PA mucA22*, which expresses a truncated 15.8-kDa MucA protein, and a *mucA* deletion mutant (Δ*mucA*). Surprisingly, only the Δ*mucA* strain was sensitive to 1 mM EDTA.

### P_*PA*4455_-*lacZ* reporter activity is affected by treatment with EDTA but not ${\text{A-NO}}_{2}^{-}$, SDS or polymyxin B

**Figure 8:**
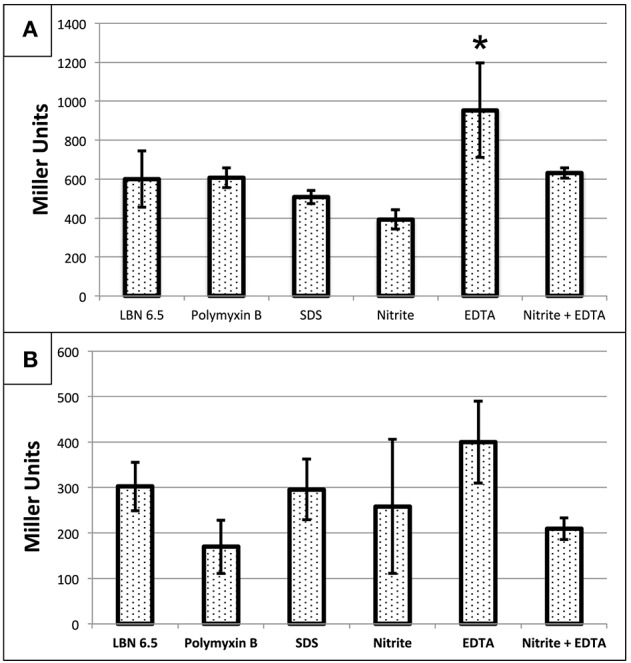
**Transcriptional activity of ***PA4455*** in response to different stressors under (A) aerobic or (B) anaerobic conditions**. PAO1 *attB*::p*PA4455-lacZ* was used to determine the activation of PA4455 under membrane stress. Cells were incubated aerobically **(A)** or anaerobically **(B)** overnight with the different stresses (10 mM NaNO_2_, 0.5 mM EDTA, 0.5% SDS, or 2 μg/ml of polymyxin B). Then, the samples were diluted to an O.D._600_ of approximately 0.4 and β-galactosidase activity was measured. Reported values are the average of 3 independent experiments. (^*^) designates >90% confidence.

We next elected to assess whether ${\text{A-NO}}_{2}^{-}$, EDTA, SDS, or polymyxin B (PMB) affected transcription of the *PA4455* gene. Wild-type bacteria were transformed with a mini-CTX-p*PA4455*-*lacZ* reporter plasmid for *attB* integration. For p*PA4455*, it was found that reporter activity was increased in response only to EDTA, but interestingly not to any of the other agents (Figure 8). This suggests that the protein is at least partially responsible for structural stability (or the response to repair) of the cell membrane. The most dramatic response was due to EDTA, which caused a ~1.6-fold increase in reporter activity. Statistical significance only existed for EDTA aerobically (*p* < 0.05 against all groups except the combination treatment) but not anaerobically using a one-way ANOVA.

### B-band but not A-band lipopolysaccharide (LPS) O-antigen mutants are defective in anaerobic growth and are more susceptible to the effects of ${\text{A-NO}}_{2}^{-}$ and EDTA

**Figure 9:**
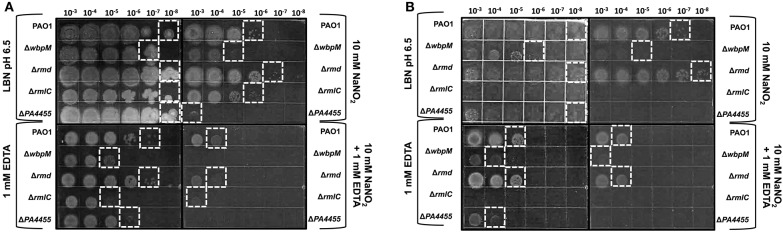
**Susceptibility of planktonic LPS mutants vs. ***PA4455*** to ${\text{A-NO}}_{2}^{-}$ and EDTA under aerobic vs. anaerobic conditions**. PAO1 (A+, B+), *wbpM* (A+, B−), *rmd* (A−, B+), and *rmlC* (A−, B−) were grown aerobically overnight at 37°C in LB, and then sub-cultured into LBN pH 6.5 media at a 1:100 ratio. Serial dilutions were spotted on LBN plates containing control 50 mM potassium phosphate, pH 6.5, ± 10 mM NaNO_2_, ± 1 mM EDTA and incubated aerobically (**A**, 24–48 h) or anaerobically (**B**, 72 h) at 37°C. The hatched white boxes designate where noticeable growth occurred on the plates.

We next postulated that one cellular structure that might be affected by the combined microbicidal activities of ${\text{A-NO}}_{2}^{-}$ and/or EDTA would be the LPS of *PA*. EDTA is notorious for damaging the outer membrane of Gram-negative bacteria such as *PA* (Alakomi et al., 2006). With such damage, LPS molecules with associated proteins have been shown to be released into the medium (Matsushita et al., 1978), further compromising the outer membrane function that can lead to cell death. One function of LPS, the major component of the outer membrane, is to help protect the membrane from potential damaging agents. We postulated that an absence of the putative ABC transporter permease, *PA4455*, could perturb the inner membrane of *PA*, that may, in turn, affect the overall behavior of the outer membrane and associated LPS molecules. Given that lipid A mutants cannot be constructed because such mutations are lethal, we used O-antigen (A-band- and/or B-band-deficient) mutants of the LPS layer and assessed the antimicrobial activity of ${\text{A-NO}}_{2}^{-}$ ± EDTA in comparison to sensitivity of wild-type bacteria. These mutants included *rmlC* (A^−^B^−^, encoding a C3′ and C5′ carbohydrate epimerase Rahim et al., 2000; Dong et al., 2007), *rmd* (A^−^B^+^, that converts GDP-d-mannose to GDP-d-rhamnose (Rocchetta et al., 1998b), and *wbpM* (A^+^B^−^; Creuzenet and Lam, 2001), respectively (Supplementary Figure 2, Figure 9). Our results revealed that an *rmd* mutant was the least affected when growing anaerobically or in the presence of either 15 mM ${\text{NO}}_{2}^{-}$ ± 1 mM EDTA (Figure 9). However, consistent with a previous report (Murphy et al., 2014), the *rmd* mutant grew more efficiently than wild-type bacteria under both aerobic and anaerobic conditions. In contrast, mutants that do not produce B-band LPS, *wbpM*, and most pronounced in *rmlC*, either grow extremely poorly or do not grow under anaerobic conditions. The *rmlC* mutant, however, was the most susceptible when grown in the presence of either 15 mM ${\text{NO}}_{2}^{-}$ or 1 mM EDTA. These observations suggest that B-band LPS is required to shield the PA outer membrane from the bactericidal effects of EDTA and A-NO_2_, yet also is essential for optimal anaerobic growth and survival (Figure 9).

### Antibiotic MIC determinations

Since the *PA4455* protein is predicted to facilitate transport of small molecules into *PA*, we next assessed whether the *PA4455* mutant possessed altered minimal inhibitory concentration (MIC) values to a large panel of antibiotics. The *PA4455* mutant was 4-, 6-, 8-, and 8-fold more sensitive than wild-type bacteria to chloramphenicol, colistin, tigecycline, and doxycycline, respectively (Table 2). In contrast, the mutant strain was 4- and 64-fold more resistant to ticarcillin/clavulanic acid and gentamicin (serving as the positive resistance control), respectively.

**Table 2:** **MIC's in μg/ml for select antimicrobial agents representing different classes of antibiotics**.

**Antimicrobial**	**PAO1**	***PA4455***	***PA4455*/pUCP-*PA4455***	**Fold resistance (R) or susceptible (S)**
Cefoxitin	>256	>256	>256	No difference
Ceftazadime	2	2	2	No difference
Cefepime	1.5	1.5	1.5	No difference
Ticarcillin/ Clavulanic Acid	32	128	96	4^R^
Pipercillin/ Tazobactam	3	4	4	No difference
Imipenem	2	2	2	No difference
Meropenem	1	1.5	1	No difference
Aztreonam	2	3	4	No difference
Amikacin	4	4	4	No difference
Gentamicin	3	192	128	64^R^
Ciprofloxacin	0.19	0.064	0.19	
Doxycycline	32	4	16	8^S^
Tigecycline	24	3	16	8^S^
Colistin	3	0.5	2	6^S^
Chloramphenicol	24	6	16	4^S^

### Attenuated virulence of a *PA4455* mutant relative to wild-type bacteria

**Figure 10:**
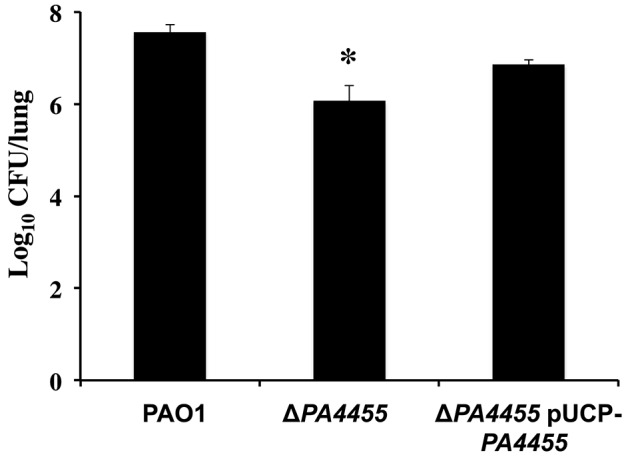
**Virulence of the PA4455 mutant is reduced relative to wild-type bacteria in a mouse acute respiratory model**. BALB/c mice (6 per bacterial strain) were infected with 1 × 10^7^ CFU of the *PA4455* mutant, PAO1, or the complemented mutant intratracheally. After 20 h of infection, the lungs were harvested and homogenized, and the resulting serially diluted suspensions were assessed for CFU. (^*^) designates × > 95% confidence interval.

Finally, we assessed the role of *PA4455* in animal virulence using an established acute murine lung infection model of *PA* infection. Mice were infected intranasally as we have previously described (Lau et al., 2005) using 1 × 10^6^ CFU of the wild-type, the *PA4455* mutant, and *PA4455*-pUCP-*PA4455* bacteria and the lungs harvested and homogenized after 20 h. The *PA4455* mutant was found to be ~10-fold less virulent than wild-type bacteria (Figure 10).

## Discussion

### Microaerobic/anaerobic bacterial biofilms in CF airway disease

This study stemmed from two seminal works in 2002 demonstrating that *PA* grows as microaerobic or anaerobic biofilms in thick mucus plugs lining the CF airway lumen (Worlitzsch et al., 2002; Yoon et al., 2002) and likely that of the COPD airway mucus (Hassett et al., 2014). Although initially the aforementioned studies were met with some skepticism (Alvarez-Ortega and Harwood, 2007), there have been 205 citations in PUBMED when the words “anaerobic” and “cystic fibrosis” were searched prior to the submission of this work for publication (February 5, 2016). The definitive supporting work was provided by a research group (Tunney et al., 2008) who reported the isolation of obligate anaerobes in CF sputum. Furthermore, *PA* infected sputum samples produce a gaseous product of anaerobic respiration nitrous oxide (N_2_O), further evidence that *PA* is undergoing this process in the CF airway soccerball-shaped biofilms (Kolpen et al., 2014).

### ${\text{A-NO}}_{2}^{-}$: relevant genes involved in resistance and translational potential

Relatedly, one hallmark of both chronic CF and COPD is the emergence of the mucoid, *mucA22* form of *PA* that are highly resistant to conventional antibiotics used to treat the aforementioned diseases. Surprisingly, Hassett (1996) has previously shown that anaerobic conditions promote mucoid stability, while static aerobic growth allows for mucoid-to-nonmucoid conversion. In 2006, we demonstrated that mucoid *mucA22* bacteria are surprisingly and uniquely susceptible to the NO-donor, ${\text{A-NO}}_{2}^{-}$ at pH 6.5, the approximate pH of the CF airway mucus (Coakley et al., 2003; Yoon et al., 2006). Specifically, we demonstrated that ${\text{A-NO}}_{2}^{-}$ killed mucoid *PA* in (a) anaerobic biofilms; (b) sterile ultrasupernatants of airway secretions derived from explanted CF lungs; and (c) a mouse model of PA lung infection in a pH-dependent fashion, with no organisms remaining after daily exposure to ${\text{A-NO}}_{2}^{-}$ for 16 days (Yoon et al., 2006). The ${\text{A-NO}}_{2}^{-}$ translational approach for the treatment of mucoid *PA* is also effective at killing methicillin-resistant *S. aureus* (MRSA) and *Burkholderia cepacia*, that, with *PA*, represent two other major CF pathogens (Major et al., 2010). Finally, Yoon et al. (2006) clearly showed that ${\text{A-NO}}_{2}^{-}$ at concentrations up to 300 mM did not elicit LDH release from human CF airway epithelia or alter transepithelial short circuit current, transepithelial water flux, or IL-8 release. More recent comparable results to the aforementioned were also found independently by another research group (Zemke et al., 2014).

The seemingly paltry level of NO derived from ${\text{NO}}_{2}^{-}$ reduction also was found to trigger dispersion of mature, preformed biofilms (Barraud et al., 2006), likely an event that threatens bacterial viability within biofilms. Bacteria such as *PA* possess an obvious protective mechanism against NO, the respiratory NO reductase (NOR). Thus, without NOR, the bacteria grow abysmally slowly under anaerobic conditions due to high endogenous NO production (~16 μM; Yoon et al., 2007). Similar to the NO detoxifying properties of NOR, much to our surprise, we also recently discovered that the major catalase of *PA*, KatA, is produced at higher levels during anaerobic growth and is essential for optimal protection against endogenous NO and ${\text{A-NO}}_{2}^{-}$ treatment in both planktonic and biofilm culture due to its ability to “buffer” NO (Su et al., 2014). Many studies in both Gram-positive and Gram-negative bacteria have clearly shown that many ABC transporters are involved in resistance to a variety of antibiotics (Reilman et al., 2014; Pletzer et al., 2015). In one particular study, overexpression in a highly antibiotic-sensitive strain of *E. coli* of SmrA, a novel multi-drug efflux pump from *Stenotrophomonas maltophilia*, allowed for resistance to fluoroquinolones, tetracycline, doxorubicin and multiple dyes (Al-Hamad et al., 2009). Our work shows that *PA4455* is essential for optimal resistance to doxycycline (alters membrane permeability), tigecycline (alters membrane permeability), colistin (punctures cytoplasmic membrane), and chloramphenicol (transport via facilitated diffusion; Table 2). A study performed with *E. coli* revealed that an ABC transporter was essential for proper transport of lipopolysaccharide to the outer membrane (Sherman et al., 2013). Our results suggest that one of the functions of *PA4455* is to stabilize the cytoplasmic membrane as well as to control the rate of ${\text{A-NO}}_{2}^{-}$ transport into *PA* during anaerobic growth. As observed with the activation of the promoter-*lacZ* fusion, the *PA4455* mutant consistently expressed higher levels of some of the genes associated with the denitrification (anaerobic respiratory) pathway. While this in itself might suggest that the membrane would have significant alterations that could allow for greater access to nitrate or nitrite, none were immediately apparent by TEM (Supplementary Figure 3). Were such alterations to be present, they may be subtle and not easily detectable as clear physical membrane deformities, but rather structural changes or reorganizations due to a less stabilized membrane.

### EDTA is synergistic with ${\text{A-NO}}_{2}^{-}$ in the killing of *PA*

EDTA has been shown to have markedly detrimental effects on bacterial outer membranes, ultimately causing leakage of LPS and increased permeabilization to biocides (Alakomi et al., 2006). In 1980, Wood et al. (1980) demonstrated the synergistic effect of EDTA and several antibiotics including penicillin G, ampicillin, tetracycline, gentamicin, and carbenicillin on mucoid and nonmucoid *PA*. EDTA has also been shown to inhibit *PA* biofilm formation, especially under anaerobic conditions (O'May et al., 2009). Supportively, a recent study has shown that ${\text{A-NO}}_{2}^{-}$ inhibited 99% of *PA* biofilm formation on primary CF epithelial cell (Zemke et al., 2014). The same group also showed that ${\text{A-NO}}_{2}^{-}$ is synergistic at killing *PA* biofilms with the membrane disrupting agent colistimethate (polymyxin E) but inhibits the microbicidal activity of aminoglycosides (Zemke et al., 2015). In CF patients, Brown et al. (1985) showed that ${\text{NO}}_{2}^{-}$ at doses as high as 250 mM were non-toxic. Based on the phenotypic evidence found associated with our *PA4455* mutant, we believe that the gene product is important for the overall membrane stability associated with *PA*. It may be possible that without *PA4455*, the membrane integrity is perturbed, thereby allowing the entrance of small molecules such as ${\text{NO}}_{2}^{-}$ that are normally not permitted full entry into the cell. This could explain the elevated transcriptional regulation of genes associated with anaerobic respiration in the *PA4455* mutant.

### B-band LPS is necessary for anaerobic growth and optimal resistance to ${\text{A-NO}}_{2}^{-}$ and EDTA

It has previously been shown that B-band LPS production is significantly reduced during anaerobic growth, while it is dramatically overproduced under oxidative stress conditions (e.g., exposure to H_2_O_2_; Sabra et al., 2003). The highly negatively charged form of B-band O antigen, known as B-band LPS, is necessary for formation of membrane vesicles inducible by oxidative stress in the form of H_2_O_2_ (Macdonald and Kuehn, 2013). Our results, unexpectedly, suggest that B-band LPS is critical not only for anaerobic growth of *PA*, but also for optimal resistance to EDTA and ${\text{A-NO}}_{2}^{-}$. The molecular basis underlying this phenomenon is currently under study. In *E. coli*, a mutant lacking a *PA4455* ABC transporter permease homolog, *MlaE*, was found to be exquisitely susceptible to EDTA/SDS and this was thought be to due to increased cellular permeabilization (Malinverni and Silhavy, 2009). One unique feature of the ${\text{A-NO}}_{2}^{-}$ and/or EDTA sensitivity of the various mutant strains used in this study was the consistent and very dramatic sensitivity of B-band LPS mutants.

## Summary

In summary, the discovery of *PA4455* as being a critical component in resistance to both ${\text{A-NO}}_{2}^{-}$ and EDTA was unexpected, yet potentially intriguing for translational purposes. Given the promising results of this study, we believe that the combination of ${\text{A-NO}}_{2}^{-}$ and EDTA represents a novel combinatorial, non-toxic treatment that could potentially achieve the translational goal of eradicating nonmucoid and mucoid *PA* (and potentially other organisms) in the CF and COPD airways.

## Author contributions

Substantial contributions to the conception or design of the work; or the acquisition, analysis, or interpretation of data for the work (CM, SS, WP, GL, TB, KC, AP, SK, JM, JL, DM, DH); drafting the work or revising it critically for important intellectual content (CM, SS, WP, GL, TB, KC, AP, SK, JM, JL, DM, DH); final approval of the version to be published (CM, SS, WP, GL, TB, KC, AP, SK, JM, JL, DM, DH); Agreement to be accountable for all aspects of the work in ensuring that questions related to the accuracy or integrity of any part of the work are appropriately investigated and resolved (CM, SS, WP, GL, TB, KC, AP, SK, JM, JL, DM, DH).

## Funding

This work was supported primarily by a Merit Review Grant from the Department of Veteran's Affairs, Cincinnati VA Hospital (Cincinnati, OH). Additional support was from ARCH Biopartners, Inc. (Toronto, Canada), Cystic Fibrosis Research, Inc. (Mountain View, CA), Cure Finders, Inc. (Sevierville, TN), Aires Pharma (San Diego, CA), and the Daniel Tyler Health and Education Foundation (Fort Collins, CO).

### Conflict of interest statement

The authors declare that the research was conducted in the absence of any commercial or financial relationships that could be construed as a potential conflict of interest. The authors received Research funding from Arch Biopartners Inc. to help conduct the study. DH and DM are insiders and have an equity position in Arch Biopartners Inc. DH has an issued patent (US 8,557,300 B2) and provisional patent application pertaining to the submitted work.

## Supplementary material

Supplementary Figure 1 **qPCR results of wild-type and mutant bacteria**. RNA was extracted from late log phase growing organisms (PAO1, *PA4455*, and pUCP-*PA4455*), then subjected to **(A)** PCR and **(B,C)** RT-PCR. Primers against the middle regions of each shown gene were used. For RT-PCR, the reported values are fold increases for each gene in each mutant bacteria over values for PAO1 (2${}^{{\Delta \Delta }_{{\text{C}}_{\text{T}}}}$). Initial ΔC_T_ measurements were calculated using each gene in the *PA4455* operon compared to the housekeeping gene, *acpP*. **(B)** Aerobic- and **(C)** anaerobically grown organisms were both used for RT-PCR, and the averages of each samples 2${}^{{\Delta \Delta }_{{\text{C}}_{\text{T}}}}$ values are reported.

Supplementary Figure 2 **Diagrams of the LPS profile of wild-type, *rmlC, rmd*, and *wpbM* mutant strains used in this study**. Each sugar moiety is given in a short table inserted within the figure. OSA, O-specific antigen; CSA, cell surface antigen. Jagged black lines in the outer membrane (gray), lipid A. Adapted from Lam et al. (2011).

Supplementary Figure 3 **TEM of wild-type, *PA4455* mutant and *PA4455* mutant complemented strains during anaerobic growth**. Sections were scanned for areas with predominant bacterial density, and images were taken at 100,000x magnification. **(A)** PAO1, **(B)** *PA4455* mutant, **(C)** *PA4455* mutant complemented with pUCP-*PA4455*.
