# Supplementary figures and images for: A Putative ABC Transporter Permease Is Necessary for Resistance to Acidified Nitrite and EDTA in Pseudomonas aeruginosa under Aerobic and Anaerobic Planktonic and Biofilm Conditions
Source: Front Microbiol. 2016 Apr 1;7:291. doi: 10.3389/fmicb.2016.00291 (PMC4817314; doi:10.3389/fmicb.2016.00291)

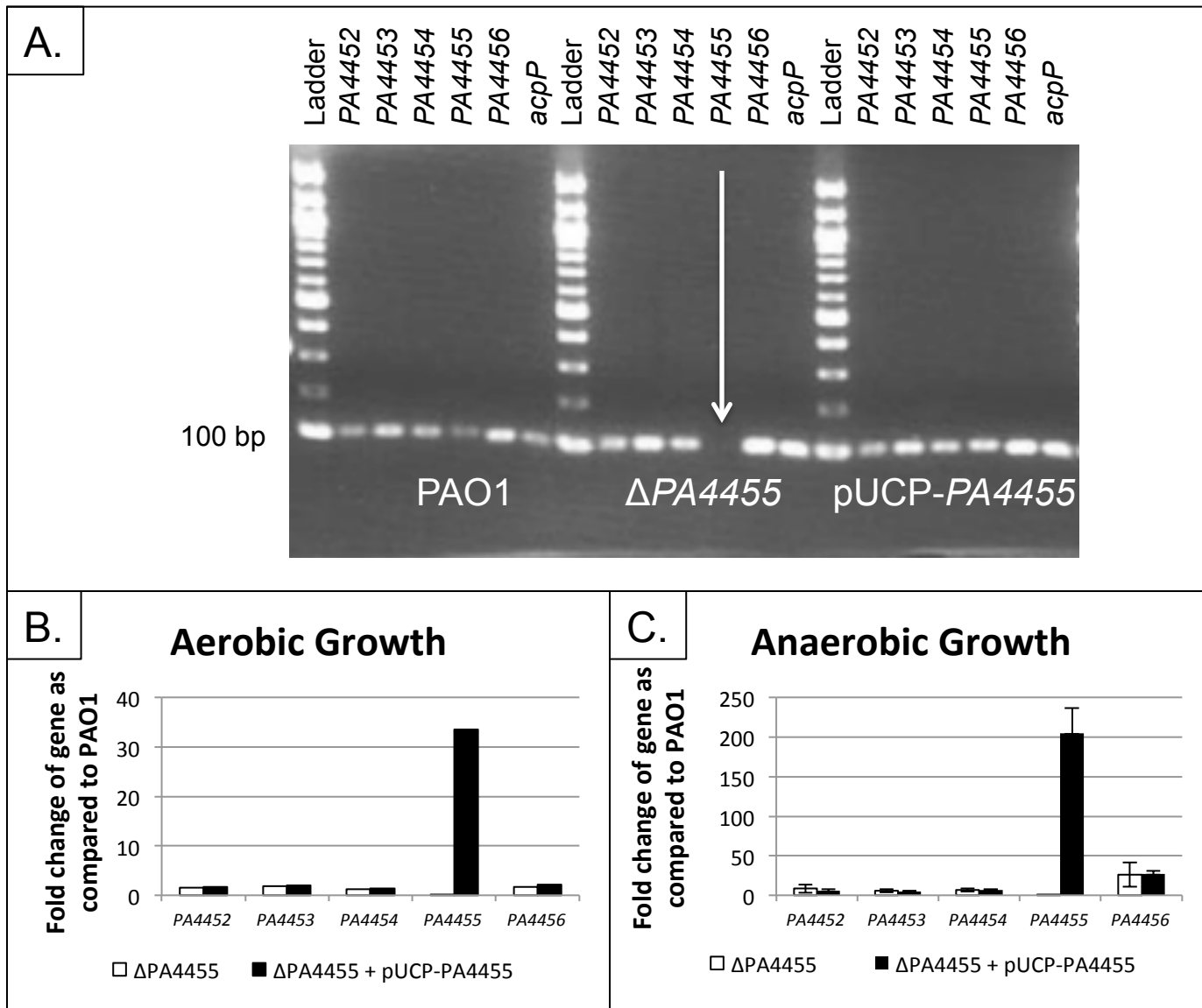

Supplement: Supplementary Figure 1 — qPCR results of wild-type and mutant bacteria. RNA was extracted from late log phase growing organisms (PAO1, PA4455, and pUCP-PA4455), then subjected to (A) PCR and (B,C) RT-PCR. Primers against the middle regions of each shown gene were used. For RT-PCR, the reported values are fold increases for each gene in each mutant bacteria over values for PAO1 (2ΔΔCT). Initial ΔCT measurements were calculated using each gene in the PA4455 operon compared to the housekeeping gene, acpP. (B) Aerobic- and (C) anaerobically grown organisms were both used for RT-PCR, and the averages of each samples 2ΔΔCT values are reported. [file Image1.pdf]

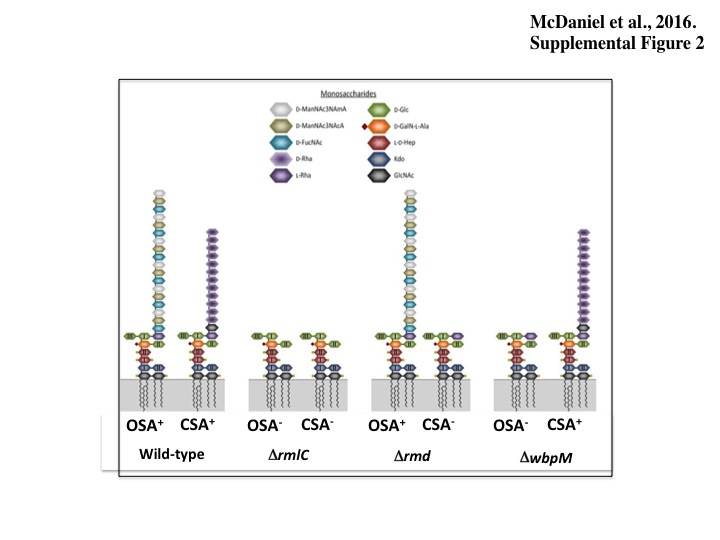

Supplement: Supplementary Figure 2 — Diagrams of the LPS profile of wild-type, rmlC, rmd, and wpbM mutant strains used in this study. Each sugar moiety is given in a short table inserted within the figure. OSA, O-specific antigen; CSA, cell surface antigen. Jagged black lines in the outer membrane (gray), lipid A. Adapted from Lam et al. (2011). [file Image2.JPEG]
