# Supplementary material for: A Putative ABC Transporter Permease Is Necessary for Resistance to Acidified Nitrite and EDTA in Pseudomonas aeruginosa under Aerobic and Anaerobic Planktonic and Biofilm Conditions
Source: Front Microbiol. 2016 Apr 1;7:291. doi: 10.3389/fmicb.2016.00291 (PMC4817314; doi:10.3389/fmicb.2016.00291)
Supplement: Supplementary Figure 3 — TEM of wild-type, PA4455 mutant and PA4455 mutant complemented strains during anaerobic growth. Sections were scanned for areas with predominant bacterial density, and images were taken at 100,000x magnification. (A) PAO1, (B) PA4455 mutant, (C) PA4455 mutant complemented with pUCP-PA4455. [file Image3.pdf]

Supplementary Figure 3

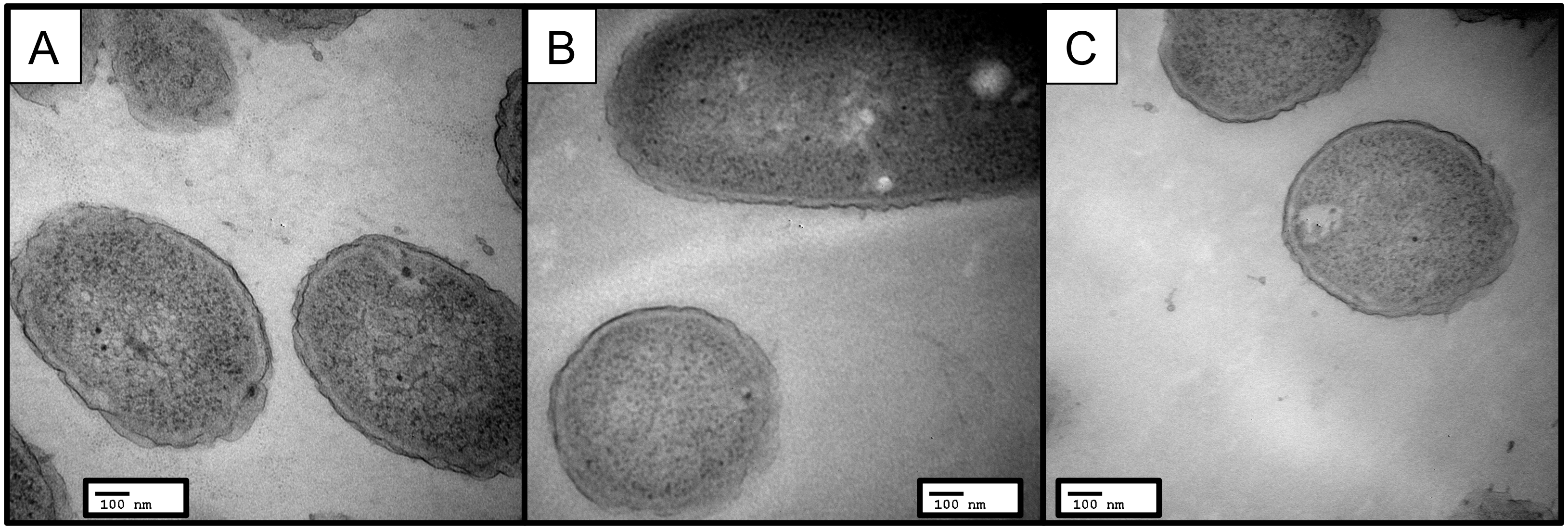

PAO1

Δ*PA4455*

Δ*PA4455* + pUCP-*PA4455*

All images at 100,000X Magnification, no noticeable difference in membrane integrity, thickness, or periplasmic size
